# The role of discounts and loss leaders in medicine procurement in Austrian hospitals - a primary survey of official and actual medicine prices

**DOI:** 10.1186/1478-7547-11-15

**Published:** 2013-07-04

**Authors:** Sabine Vogler, Nina Zimmermann, Claudia Habl, Jan Mazag

**Affiliations:** 1 WHO Collaborating Centre for Pharmaceutical Pricing and Reimbursement Policies, Health Economics Department, Gesundheit Österreich GmbH / Österreichisches Bundesinstitut für Gesundheitswesen (GÖG/ÖBIG, Austrian Health Institute), Vienna, Austria; 2 Statny Ustav pre Kontrolu Lieciv (SUKL, State Institute for Drug Control), Bratislava, Slovakia

**Keywords:** Medicine prices, Pharmaceutical policy, Hospital, Discount, Cost-free medicines, Austria

## Abstract

**Background:**

Knowledge about the prices of medicines used in hospitals, particularly the actually achieved ones, is scant. There are indications of large discounts and the provision of medicines cost-free to Austrian hospitals. The study aims to survey the official and actual prices of medicines procured by Austrian hospitals and to compare them to the out-patient prices.

**Methods:**

Primary price collection of the official hospital list prices and the actually achieved prices for 12 active ingredients as of the end of September 2009 in five general hospitals in Austria and analysis of the 15 most commonly used presentations.

**Results:**

The official hospital list prices per unit differed considerably (from 1,500 Euro for an oncology medicine to 0.20 Euro for a generic cardiovascular medicine). For eight on-patent medicines (indications: oncology, anti-inflammatory, neurology-multiple sclerosis and blood) actual hospital medicine prices equaled the list prices (seven medicines) or were lower (one medicine) in four hospitals, whereas one hospital always reported higher actual prices due to the application of a wholesale mark-up. The actual hospital prices of seven medicines (cardiology and immunomodulation) were below the official hospital prices in all hospitals; of these all cardiovascular medicines were provided free-of-charge. Hospital prices were always lower than out-patient prices (pharmacy retail price net and reimbursement price).

**Conclusion:**

The results suggest little headroom for hospitals to negotiate price reductions for “monopoly products”, i.e. medicines with no therapeutic alternative. Discounts and cost-free provision (loss leaders) appear to be granted for products of strategic importance for suppliers, e.g. cardiovascular medicines, whose treatment tends to be continued in primary care after discharge of the patient.

## Background

In European countries pharmaceutical policies have usually been addressed to the out-patient sector which is also reflected in literature about system descriptions, analyses and evaluations [1-7]. Since pharmaceutical expenditure in hospitals has been fairly constant over the years (usually 5-10% of a nation’s medicines budget), it has for a long time not been a priority of policy makers [8]. Research on the hospital sector focused on identifying costs of clinical events, economic evaluations of alternative interventions as well as methodology work for exploring elements of hospital costs [9-13]. However, the prices of medicines procured in hospitals have not been within the scope of research.

Recently, hospital medicines, including their price components, started to attract the attention of policy makers. This may be attributable to the growth of pharmaceutical expenditure in hospitals in the last years (e.g. introduction of new, expensive medicines). Further, it is generally acknowledged that medication started during the hospital stay can impact the future medicines prescribed after a patient has been discharged [14-21].

In Austria, the pharmaceutical sector is organized in a two-tier system with different public payers for the out-patient (i.e. community care) and the hospital sectors. Medicines prescribed for use in the out-patient sector are funded by the sickness funds; the decision about their inclusion in the reimbursement list (“Erstattungskodex”, EKO) is done after a pharmacological, medical-therapeutic and health-economic evaluation [22,23]. Medicines in hospitals are financed out of the hospital budgets and are, apart from some exemptions, included in the Diagnosis Related Groups (DRG) remuneration system. Almost half of the hospitals in Austria are so-called “Fondskrankenanstalten” (“fund hospitals”): These public and private non-profit hospitals for public benefit are financed by the Provincial Health Funds which receive funds from the federal government, the provinces, local authorities and the social insurance institutions [24]. Costs for reimbursed medicines used in out-patient care are covered by the Social Health Insurance, except for the prescription fee which is paid out-of pocket by the patient [22,23,25].

In Austria, medicine prices are regulated in specific settings: At ex-factory price level, the prices of medicines to be used in the out-patient sector are subject to price control if the manufacturer seeks reimbursement. To set the price, the method of external price referencing (international price comparison) is applied, and prices must not exceed average of the prices in other European Union Member States [22]. If a company launches a medicine only in the hospital setting, then external price referencing is not applicable since there is free pricing: the manufacturer can determine the price of the medicine.

As distribution remuneration statutorily regulated wholesale mark-ups and pharmacy mark-ups are added on the ex-factory price [26]. This is primarily relevant for the out-patient sector. Hospital pharmacies normally do not dispense medicines to out-patients; however five of the total of 46 Austrian hospital pharmacies also act as a community pharmacy: when they dispense to out-patients, they apply the mark-up regulation for community pharmacies. Wholesale mark-ups are of marginal importance in the in-patient sector because Austrian hospitals are predominantly supplied directly by manufacturers and not via wholesalers [24].

Hospital prices are expected to be lower since hospitals purchase medicines from the manufacturers and are supposed to achieve discounted prices compared to the medicine prices applied in the out-patient sector throughout the country. However, the actually achieved prices by hospitals are not published by the hospitals whereas the official prices applied in the out-patient sector are publicly available following the provisions for publication of the Transparency Directive [27].

In the last decades, price analysis methodology has been improved, and price surveys and comparisons both at European and international level were undertaken by commercial companies (e.g. IMS Health), international institutions such as the Organisation for Economic Co-operation and Development (OECD) and the World Health Organization (WHO) [28,29], as well as by academics [30-32]. Further, in many European countries public authorities responsible for pharmaceutical pricing established their own systems of price surveys which is needed when they compare to the prices of other countries in setting the price of a medicine in their country [33]. Austria, which, as described, applies external price referencing, also established a national price survey system. Based on a regulation in the Austrian General Social Insurance Law [34], the Austrian Health Institute runs the national “Pharma Price Information” (PPI) service of medicine prices in all EU Member States which supports the Austrian Pricing Committee which is in charge of calculating the EU average price [35].

Despite these advances in price analyses and comparisons, to the best knowledge of the authors, all price comparisons available in English or German are, with one exemption, limited to out-patient medicine prices. We are aware of only one unpublished European price survey for hospital medicines which, commissioned by the Danish Ministry of Health, was undertaken for eight countries (Canada, Denmark, England, France, Germany, Norway, the Netherlands, Sweden) (COWI: Analysis of hospital pharmaceuticals, unpublished). For Austria, no survey of medicine prices in the in-patient sector has been published yet.

This study aims to address this gap by surveying the prices of medicines used in Austrian hospitals. The article will report both the official list prices and the actual prices achieved in medicine procurement in hospitals and compare them to the prices applied in the out-patient sector.

## Methods

The methodology for the price survey was elaborated in the framework of the European Commission co-funded project PHIS (Pharmaceutical Health Information System) which aimed to improve knowledge about in-patient and out-patient pharmaceutical systems in all EU Member States [36]. The PHIS activities included a price comparison of medicine prices in hospitals of five selected countries (Austria, the Netherlands, Norway, Portugal, and Slovakia). Therefore, we developed a methodology for price collection and international comparison of hospital medicine prices (Vogler S, Habl C, Leopold C, Morak S, Mazag J, Zimmermann N: PHIS Hospital Pharma Methodology Paper; unpublished). The methodology was elaborated by the authors in spring 2009, revised after feed-back of the PHIS project management team and the project Advisory Board in summer 2009.

### Definitions

This paper uses the terminology as defined in the glossary on pharmaceutical terms developed by the WHO Collaborating Centre for Pharmaceutical Pricing and Reimbursement Policies [37]. Definitions of price types are explained in the text or below the tables and graphs.

### Selection of medicines

A multi-phase approach was applied for the selection of the molecules for the price data collection. We only considered medicines which were authorized for the Austrian market. We focused our sample on active ingredients which account for a comparatively high share within the hospitals’ budgets. For identifying the active ingredients accounting for the highest expenditure in the in-patient sector in 2007 or 2008 (when available), we analyzed the Top 10 lists of the five countries included in the PHIS price comparison, which were provided by country experts, mostly from public authorities [24,38-41].

The molecules of this draft sample were checked to see if they complied with the criteria of “commonly used”, “standard therapeutic groups” and “high diagnostic relevance”. Additionally, the sample was extended by medicines which might impact the pharmaceutical bill of the countries due to high consumption (e.g. high volume products in the out-patient sector such as cardiovascular medicines). Based on this selection process, the following groups were taken into consideration: cardiovascular medicines, contrast media, hematology medicines, neurology medicines (especially for Multiple Sclerosis), nutrition (electrolyte), oncology medicines (breast, colon, leukemia), and transfusion medicines.

Furthermore, we looked at which active ingredients were investigated in other projects. Three price surveys were considered as relevant reference work: the COWI report (COWI: Analysis of hospital pharmaceuticals, unpublished), the voluntary internal price exchange exercise INFOPRICE among EU Member States in the framework of the Transparency Committee, and an orphan medicines survey (Habl C, Bachner F: Orphan medicines survey, unpublished). We aimed to achieve a balance between the on-patent and off-patent segments. For methodological reasons we decided to disregard medicines whose prices were difficult to compare, e.g. due to different dosage forms or bulk packages in the different countries.

As a result, we compiled a list of 20 active ingredients. However, we performed the survey on the first 12 ranked active ingredients only (see Table 1) because we did not want to burden the hospital staff with a time-intensive survey.

**Table 1:** List of active ingredients selected for the survey

**No.**	**Active ingredient**	**ATC code**	**Key therapeutic area**
1	Trastuzumab	L01XC03	oncology
2	Docetaxel	L01CD02	oncology
3	Rituximab	L01XC02	oncology
4	Etanercept	L04AB01	rheumatoid arthritis
5	Imatinib	L01XE01	orphan/oncology
6	Immunoglobulin	J06BA02	immunomodulation
7	Infliximab	L04AB02	anti-inflammatory
8	Interferon beta-1α	L03AB07	neurology-multiple sclerosis
9	Amlodipin	C08CA01	cardiology
10	Simvastatin	C10AA01	cardiology
11	Atorvastatin	C10AA05	cardiology
12	Clopidogrel	B01AC04	blood

### Selection of hospitals

We undertook the price survey in five hospitals in five different Austrian provinces. All establishments are general hospitals, and they are considered as public hospitals (4 are owned by the provinces, and one is a non-profit hospital owned by a religious institution). Table 2 describes the main characteristics of the five selected hospitals compared to the hospitals throughout the country. In many respects the hospitals of the sample have identical or similar features as most hospitals in Austria. A major difference, however, regards the hospital pharmacy which four of the five sample hospitals run; this is only the case in less than 20% of Austrian hospitals [24]. The majority of hospitals in Austria, and also one hospital of this price survey, organise their medicines supply management via a “pharmaceutical depot” which is a unit within the hospital for the internal supply of the hospital with medicines. A “pharmaceutical depot” usually has fewer tasks, competences and responsibilities than a hospital pharmacy, and it might be run by the hospital pharmacy of another hospital [24].

**Table 2:** Characteristics of the hospitals included in the survey

**Parameter**	**Hospitals of the price survey**	**Hospitals in total in Austria**
Number of hospitals	5	266 (December 2008)
Type of hospitals and geographic distribution	All are general hospitals; 1 in Vienna, 1 in the South East of Austria, 1 in the South, 1 in the North Western part, 1 in the biggest province of Austria	38% of all hospitals were general hospitals (2007)
Ownership	All are public hospitals; 4 are owned by Austrian provinces, 1 is in the ownership of a non-profit religious congregation	Around 60% of hospitals are public
Size of hospitals	4 are big hospitals (> 500 acute care beds);	36 hospitals with more than 400 acute care beds
	One hospital is middle sized (between 400 and 500 acute care beds)	
Hospital pharmacy	4 hospitals have a pharmacy;	17% of all hospitals have a hospital pharmacy (2008). The other, often smaller hospitals are equipped with a pharmaceutical depot
	1 hospital has a “pharmaceutical depot” which is delivered by a wholesaler with an affiliated pharmacy^1^	
Purchasing policies	Negotiations are the key policy for purchasing medicines in the 5 hospitals.	Same situation for all hospitals in Austria – tendering of medicines is only done in rare cases, but a rising trend can be observed
	Tendering by hospitals is of minor importance in 4 hospitals;	
	1 hospital commissioned a wholesaler following a tendering process	
Level of centralisation in purchasing	Decentralised purchasing (purchasing at hospital level or at the hospital owner level)	Same situation for all hospitals in Austria
Pharmaceutical expenditure in % of total hospital expenditure	Around 7% of the hospital expenditure	Around 9% of the hospital expenditure

^1^ Pharmaceutical depots are only allowed to purchase the required medicines from another licensed pharmacy in the European Economic Area (EEA).

Source: Reference data on Austria in total from [15].

### Price survey methodology

We collected the price data and other required information during study visits to the Austrian hospitals. For doing so, we developed a price survey template and a questionnaire to guide through interviews.

The price survey template comprised fields on the selected products and for surveying price data as well as background information on the procurement of these medicines. We pre-filled the trade name of the selected active ingredients, the ATC code and – if possible – the manufacturer name. We asked for the official hospital list prices and the actual (negotiated) hospital prices (including and excluding value-added tax) of the medicine packs as of 30 September 2009. In the cases of off-patent medicines, with different products being available, we surveyed the most expensive product (usually the original medicine) and the least expensive one. Further, we collected additional explanatory information on discounts, rebates or other arrangements such as cost-free medicines or rebates in kind.

The questionnaire for the accompanying interviews contained questions related to the hospital and hospital pharmacy (delivery chain and pharmaceutical provision in that hospital, funding of medicines, monitoring activities by the hospital pharmacy, initiatives for a more rational use of medicines and initiatives to improve cooperation at the interface of hospital and out-patient care, for example).

On average, the study visits took about three hours per hospital. In all cases the chief/responsible hospital pharmacists of the hospital pharmacies, and in one case a “pharmaceutical depot”, personally provided the price data and were available for the interview. In return for providing data, the hospital pharmacists were ensured anonymity.

### Price analysis

The data collection yielded a wide variety of presentations of different pharmaceutical forms, strengths and pack sizes. For each active ingredient we identified one specific product in a common pharmaceutical form, strength and pack size which we compared to a medicine of the identical or similar pharmaceutical form, strength and pack size in the other hospitals. The price of a medicine in a specific form and package size with most reported prices in the hospitals (defined as “most commonly used” presentations) was selected for the analysis. Even if we had collected prices of many more presentations (e.g. different pack sizes), these were excluded due to missing reference products in the other hospitals.

Hospital prices were analyzed as net prices, i.e. excluding the value added tax, and as “unit prices” (i.e. per tablet, vial) to allow for a comparison in case of different pack sizes (e.g. 28 and 30 tablets). Possible rebates granted by specific companies ex-post, e.g. at the end of a year if a certain volume had been purchased, could not be taken into account.

The relevant comparator price types applicable for medicines used in out-patient care were the ex-factory price, the pharmacy retail price net and the reimbursement price (“Kassenpreis”) as of 30 September 2009: The pharmacy retail price is applicable at community pharmacy level, and the reimbursement price is the price which Austrian sickness funds pay for the medicine in the out-patient sector. It is lower than the pharmacy retail price net.

## Results

### Availability

For seven of the 15 medicines surveyed, only the original products were available on the Austrian market. Where generic alternatives were available, in two cases (simvastatin, immunoglobulins) all hospitals applied generic presentations, in one case (atorvastatin) all hospitals used the original products, and in the case of amlodipidin four of the five hospitals opted for the original products. Two competitors for Interferon beta-1α were on the market and both were used in three of the five surveyed hospitals. As a result, 15 presentations in an identical pharmaceutical form – for the sake of readability referred to hereafter as “products” or “medicines” – were subject to the analysis.

**Figure 1:**
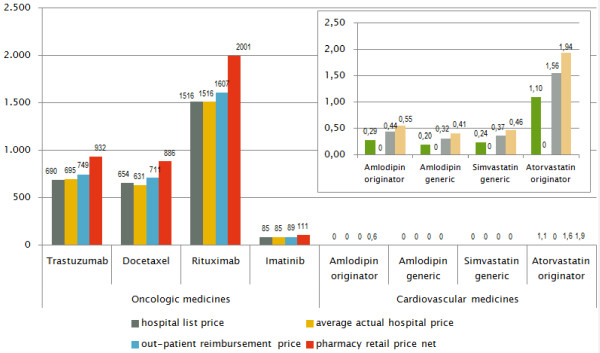
**Price differences of oncology and cardiovascular medicines between in the hospital and out-patient sectors.**

Table 3 presents the prices expressed per unit for the 15 products in the surveyed hospitals compared to the out-patient sector. The ex-factory prices for medicines in out-patient use are not indicated separately as the official hospital prices correspond to them. For two indications which were represented by a higher number of medicines in the sample (oncology, cardiology) the price data results are separately presented in Figure 1.

**Table 3:** Prices (per unit) for 12 active ingredients in Austrian hospitals and in out-patient care

**Active ingredient**	**O/G**^**1**^	**Hospitals (n = 5 unless otherwise specified), range**		**Out-patient sector**	
		**Official list price**^**2**^	**Actual hospital price**^**3**^	**Pharmacy retail price net**^**4**^	**Reimbursement price**^**5**^
		**Prices as of 30 September 2009**			
**Oncology**					
Trastuzumab 150 mg powder for concentrate for solution f. infusion	O	690.-	690.- – 713.81	932.18	748.60
Docetaxel 1 × 2 ml 80 mg/2 ml concentrate and solvent for solution for infusion	O	654.06	622.90 – 663.67	885.68	711.30
Rituximab 500 mg concentrate and solvent for solution for infusion	O	1516.43	1516.43	2001.36	1607.30
Imatinib 400 mg tablets (30 tabs)	O	84.87	84.87 (n = 4)	111.12	89.24
**Cardiology**					
Amlodipin 5 mg tablets (28 units)	O	0.29	0.00 (n = 4)	0.55	0.44
Amlodipin 5 mg tablets (28 units)	G	0.20	0.00 (n = 1)	0.41	0.32
Simvastatin 20 mg tablets (30 units)	G1	0.26	0.00 (n = 2)	0.49	0.40
Simvastatin 20 mg tablets (30 units)	G2	0.23	0.00 (n = 3)	0.45	0.40
Atorvastatin 20 mg tablets (30 units)	O	1.10	0.00	1.94	1.56
**Rheumatoid arthritis**					
Etanercept 1 ml (50 mg/ml) solution for injection in a prefilled pen	O	242.53	242.53 (n = 2)	323.65	259.93
**Immunomodulation**					
Immunoglobulin 100 mg/ml, solution for injection in vial (50 ml)	G1	246.18	185.- – 190.- (n = 4)	348.36	279.20
Immunoglobulin 100 mg/ml, solution for injection in vial (50 ml)	G2	219.40	160.- – 185.-	310.45	248.85
**Anti-inflammatory**					
Infliximab 100 mg powder for concentrate for solution for infusion	O	565.50	565.50 (n = 4)	762.32	612.20
**Neurology-multiple sclerosis**					
Interferon beta-1α 1 × 0.5 ml (44 mcg/0.5 ml) solution for injection – prefilled pen	O	81.25	81.25 (n = 3)	107.68	86.48
**Blood**					
Clopidogrel 75 mg tablets (28 units)	O	1.64	1.64 – 1.71	2.90	2.32

^1^*O* original product, *G* generic; in case of more than one generic, numbering of the generics (G1, G2).

^2^ Equals the ex-factory price for out-patient medicines. No range, as this is the same in all hospitals.

^3^ The (actual) price or amount paid by a hospital (or hospital pharmacy) in order to take delivery of certain unit of medicines. The price excludes the value added tax (VAT).

^4^ The price charged by retail pharmacies to the general public. It includes any pharmacy mark-up or dispensing fee. The net price excludes VAT.

^5^ Reimbursement price paid by Austrian sickness funds, called “Kassenpreis”. However, seven of the selected products are not included in the out-patient reimbursement list (“Erstattungskodex”, EKO). Therefore the indicated out-patient prices are rather of theoretic character.

### Price comparison across medicines

The sample of the 15 medicines varied from high-priced medicines (mainly in oncology) with ex-factory prices (official hospital list prices) per unit of several hundreds of Euros to low-priced medicines around 0.25 Euro (in cardiology). The highest priced medicine of the sample was rituximab 500 mg with an ex-factory price per unit of 1,515.- Euro, whereas a generic version of amlodipin 5mg (priced 0.20 Euro per tablet) was at the other end of the range.

The actual hospital prices were lower; some medicines were provided cost-free to all hospitals.

### Actual hospital prices compared to official hospital prices

In the case of seven medicines (trastuzumab 150 mg, rituximab 500 mg, imatinib 400 mg, etanercept 50 mg, infliximab 100 mg, Interferon beta-1α, clopidogrel 75 mg), the actual hospital prices equaled the hospital list prices, or they were, in one hospital, even higher than official hospital list prices due to the application of a wholesale mark-up.

For docetaxel 1×2 mg 80 mg/2 ml this hospital, supplied by a wholesaler, also reported a 2% higher actual hospital price compared to the official list price, whereas the other four hospitals had lower actual hospital prices than official list prices. Three hospitals reported receiving rebates in kind (i.e. for 20 vials they get one free).

For the remaining seven products, the actual hospital prices were lower than the official hospital prices in all hospitals: two medicines for immunomodulation (immunoglobulins 100 mg/ml in two different pharmaceutical forms) had a 15%-27% lower price. Five medicines (all cardiology medicines: amlodipin, simvastatin, atorvastatin) were provided cost-free to the hospitals, independently of whether they were original products or generics.

While for high-priced oncology medicines hospitals could not achieve any discounts, cardiology medicines of the sample were provided for free (Figure 1).

### Price range among the hospitals

As the official hospital list price corresponds to the ex-factory price, the surveyed list prices for identical medicines did not differ among hospitals.

Few differences in the actual hospital prices of identical medicines were observed among the surveyed hospitals. Differences were found for five products of the sample (trastuzumab 150 mg, docetaxel 1×2 mg 80 mg/2 ml, two presentations of immunoglobulin 100 mg/ml, and clopidogrel 75 mg). In the case of trastuzumab 150 mg, docetaxel 1×2 mg 80 mg/2 ml and clopidogrel 75 mg the actual hospital prices were identical in four hospitals whereas the fifth hospital the medicines had a higher actual hospital price.

Further, we noticed differences in the official list prices and actual hospital prices of different generic products of the same active ingredient in an identical presentation (i.e. in the same strength and pack size). This was the case for two products (simvastatin and immunoglobulins) where hospitals reported purchasing different products: For simvastatin the hospital list prices ranged between 0.23 and 0.26 Euro per unit, but all hospitals received these products for free. Hospital list prices for the immunoglobulins in the same pharmaceutical presentation differed between 219.40 and 246.18 Euro per unit depending on the purchased brand. Actual hospital prices for these products also showed price variations (between 160 and 190 Euro) among hospitals, but the actual prices were always lower than the list prices.

### Hospital prices compared to out-patient prices

The surveyed pharmacy retail prices net were always higher than the prices applied in the hospital setting – regardless of the surveyed price types in hospitals. Also, the out-patient reimbursement prices (“Kassenpreis”) always exceeded the hospital prices.

As shown in Figure 1, the smallest difference between the official hospital list price and the out-patient prices was observed for the high-price product rituximab 500mg (reimbursement price: +6% and pharmacy retail price: +32% compared to the hospital list price) and the largest difference for a generic version of amlodipin 5mg (reimbursement price: +58% and pharmacy retail price: +106%).

## Discussion

Our study was the first price survey undertaken for medicines used in hospitals in Austria. It was done for a sample of 12 active ingredients in a few hospitals. Even though we collected several price data points, our sample of comparable presentations (in the same pharmaceutical form, strength and pack size) was rather small, and sometimes the “most commonly used form” identified for the comparative analysis was not used in all hospitals surveyed. We acknowledge the limitations with regard to the number of products and hospitals, and we are aware that, as some of the products are only used in hospitals and not even included in the out-patient reimbursement list (“Erstattungskodex”, EKO), the comparison of the hospital prices to the out-patient prices could be considered as only of theoretical character for these products.

Despite these limitations, we believe that this price survey provides added value to the scientific community and to policy makers: Our study contributes considerably to transparency since no price survey for medicines in hospitals had been published before. Additionally, it brings attention to an area of health care which is of major relevance both economically and in terms of quality. Further, we developed and piloted a methodology which can easily be adopted and used for future surveys in Austria and other countries with a similar health care system (e.g. European Union Member States).

The development of a methodological design was a key prerequisite from a technical point of view. Another requirement was the building of trust with the hospital pharmacists, who were the data providers, and the establishment of a common understanding of the study’s rationale. In Austria, though hospital pharmacists appear to be well connected, in particular via the associations (Austrian Association of Employed Pharmacists, Austrian Association of Hospital Pharmacists), prices of medicines are not officially shared among them nor are they published, so the communication of price data to us as external people was a considerable indicator of trust.

The lack of transparency about the medicine prices paid by hospitals can, to some extent, be explained by the procurement policies in Austria. A major part of medicines is purchased in a decentralized way, mostly directly from the manufacturer, by the individual hospitals; and the price is the outcome of negotiations. There are initiatives of joint purchasing, in particular for high-cost medicines, by the hospital owner organizations. The use of tenders is still low, but increasing in Austria [24]. There is a similar pattern in the procurement of medicines for hospitals in Germany and in several Central and Eastern European countries, whereas tendering of medicines, in many cases done at central or regional level, is the key purchasing strategy in the remaining EU Member States [8,15]. Procurement based on negotiations, in particular at the level of individual hospitals, might negatively impact the transparency of prices since the purchasers are told by the suppliers that they would be granted the best prices if they in return agreed to keep them confidential. However, our results could not confirm better prices for individual hospitals since price differences among hospitals were identified in very few cases.

For the out-patient sector, well-developed and tested approaches for price surveys exist [28-31,42]. Developing the survey methodology, we had to understand if there is a price type of the out-patient sector to which the hospital list price and the actual price might correspond. Our assumption that the official hospital list price in Austrian hospitals equals the ex-factory price was confirmed by the hospital pharmacists involved in the price survey. No corresponding price type of the out-patient sector could be identified for the actual hospital price since it is a hospital specific price.

The survey of the actual prices also served to test the hypothesis of the low actual hospital prices and the provision of cost-free medicines (loss leaders) in Austrian hospitals. We found some differences between the actual hospital prices and the official prices (ex-factory prices). These price differences were, however, observed for only some, usually lower-priced medicines. In cases where price differences were identified, the actual prices were, in most hospitals, lower (or even zero), with the exception of one hospital which had higher prices for specific, mostly expensive on-patent medicines. This hospital had no hospital pharmacy; the “pharmaceutical depot” in charge of the provision of medicines in that hospital was supplied by a wholesaler. The higher price in that hospital was attributable to the wholesale mark-up which was included in their actual prices.

While the extent of discounts granted did not differ among the hospitals surveyed, the results revealed a pattern with regard to the kind of medicines: In general, no discounts could be negotiated for the high cost medicines. This is, for instance, the case for three of the four oncology medicines included in our sample, all of them on-patent. The price survey results supported findings of interviews performed with the hospital pharmacists before collecting the price data. Hospital pharmacists stated that for medicines which “really count” (i.e. accounting for a major part of the hospital pharmaceutical budget) hospitals are not successful in negotiating discounts, and they have to pay the full, i.e. ex-factory, price. The study results suggest that there appears to be little or no room for price reductions for “monopoly products”, i.e. medicines with no therapeutic alternatives.

We acknowledge that ex-post rebates were not taken into account in our price survey since they could not be assessed by the data providers at the time of the price data collection. Rebates are granted by the suppliers at the end of the year, with reference to the sales volume of a hospital. They are known to be a common commercial instrument in European countries (e.g. Portugal) [15,39], and their existence in Austria was confirmed by the pharmacists of the sample hospitals.

Price reductions in the form of discounts (i.e. reflected in the actual hospital prices) were observed whenever therapeutic alternatives were available. These were generics, but competition also appeared to be triggered by original products considered as alternatives. We observed this pattern for the immunoglobulins whose lower actual hospital prices compared to their official hospital list prices might be attributable to the choice of different products (brands) available.

In Austria a cost-free provision of medicines to hospitals is allowed and applied. This practice is forbidden in some European countries (e.g. Denmark, Hungary, Italy, Lithuania, and United Kingdom) [15], while there are no explicit provisions in some others (e.g. Bulgaria) [43]. Discounts and rebates of up to 100%, which eventually make the products cost-free, are known from a few other countries (e.g. Portugal, Slovakia) [39,40]. In our price survey on Austria, we found the provision of cost-free medicines only in one indication: cardiology. But all cardiovascular medicines of the sample, irrespective of their patent status, were supplied cost-free.

The group of medicines to treat cardiovascular diseases, which account for a high share in the disease burden [44], could be characterized as “strategic medicines” from the industry’s point of view. Cardiovascular medicines account for high volumes in the out-patient sector and thus contribute to high expenditure [45]. As the starting treatment with specific (cardiovascular) medicines by specialists impacts the future use [46], manufacturers are interested to get the product applied already in hospitals in order to initiate follow-up prescriptions in out-patient setting [47].

Prioritization is most important when resources are scarce [48]: Hospital pharmacists have to decide which medicines (high-cost medicines for few patients or a larger quantities of lower-priced medicines for larger patient populations) they will purchase given the limited budgets, and whether they agree to receive discounted and cost-free medicines in spite of the negative impact for the overall health care system. Particularly in the case of financial pressure, hospital pharmacists, who are accountable to the hospital management, are happy about any savings achieved by discounted and cost-free medicines. They are aware of the fact that with accepting free-cost medicines they impact the continuing treatment with these products after discharge of the patient (personal communication). Their procurement strategies result from dual financing, with different payers for the pharmaceutical bill in the out-patient and hospital sectors. The authors see a need for the development and implementation of effective policy options which could improve the medicines management at the interface of hospital and out-patient sector. Good practice examples from other countries (e.g. joint reimbursement lists in Stockholm County [49], or Scotland [50]) might serve as models.

The hospital sector has often been criticized for its assumed lower hospital prices compared to the out-patient sector and for cost-free medicines. Our price collection confirmed that hospital prices, even if not discounted, are always lower than out-patient retail prices – irrespective of the out-patient price type (pharmacy retail price or reimbursement price). This is attributable to the involvement of more distribution actors in the out-patient sector and their statutory remuneration for the cost for handling distribution. The different price levels in the in-patient and out-patient sectors, which is explained by these factors, is not of concern for policy makers, but the critical issue is that, via the practice of granting discounts and providing cost-free products, the treatment of patients is likely to be started with medicines which account for high volume and eventually high expenditure where medication is continued with these products in the out-patient sector.

## Conclusions

The study offers, for the first time in Austria, information about medicine prices in hospitals, and thus contributes to transparency. The suppliers’ argument of granting better prices to a hospital in return for confidentiality could not be confirmed. Discounts and rebates are granted for some, but not all medicines: The results suggest that there is no or little room for price reductions on “monopoly products”, for which no therapeutic alternatives are available. The study confirmed the practice of providing loss leaders to hospitals, particularly in strategic relevant therapeutic areas where medication is supposed to continue in the out-patient treatment. The starting treatment in a hospital with a higher priced medicine is expected to increase resource pressure in the out-patient sector, and to cause concern among patients if their therapies are switched, potentially leading to irritation and confusion with medicine regimens, particularly in the elderly.

The strategies applied by the different stakeholders (e.g. pharmaceutical industry, hospital pharmacists, payers) are influenced by the underlying organization of the Austrian health care system which is characterized by different payers in the different sectors (dual funding). The results of the price survey call for interface management initiatives to improve the coordination and cooperation between the in-patient and out-patient sectors. Policy makers are urged to develop and implement appropriate policies.

The study should be considered as a starting point to continue improving transparency about medicine prices in the in-patient sector. Further research, e.g. involving more hospitals, broadening the sample of products, is recommended.

## Competing interests

The authors declare that they have no competing interests.

## Authors’ contributions

All authors contributed to the paper’s conception, design and production. SV wrote major parts of the article. SV, NZ, CH and JM developed the methodology for the price study. NZ did the primary price collection in the Austrian hospitals and was in charge of the analysis of the data. All authors critically revised the article and have approved the final version for submission.
